# NiMoO_4_ Nanosheets Embedded in Microflake-Assembled CuCo_2_O_4_ Island-like Structure on Ni Foam for High-Performance Asymmetrical Solid-State Supercapacitors

**DOI:** 10.3390/molecules28196840

**Published:** 2023-09-28

**Authors:** Gaofeng Li, Lingling Chen, Longfei Li

**Affiliations:** 1 Institute of Advanced Energy Storage Technology and Equipment, Faculty of Mechanical Engineering and Mechanics, Ningbo University, Ningbo 315211, China; 2 Institute of Advanced Energy Storage Technology and Equipment, School of Materials Science and Chemcal Engineering, Ningbo University, Ningbo 315211, China; 2211260080@nbu.edu.cn (L.C.); 2111086089@nbu.edu.cn (L.L.)

**Keywords:** CuCo_2_O_4_, NiMoO_4_, heterostructure, micro/nanostructure, solid-state supercapacitor

## Abstract

Micro/nano-heterostructure with subtle structural design is an effective strategy to reduce the self-aggregation of 2D structure and maintain a large specific surface area to achieve high-performance supercapacitors. Herein, we report a rationally designed micro/nano-heterostructure of complex ternary transition metal oxides (TMOs) by a two-step hydrothermal method. Microflake-assembled island-like CuCo_2_O_4_ frameworks and secondary inserted units of NiMoO_4_ nanosheets endow CuCo_2_O_4_/NiMoO_4_ composites with desired micro/nanostructure features. Three-dimensional architectures constructed from CuCo_2_O_4_ microflakes offer a robust skeleton to endure structural change during cycling and provide efficient and rapid pathways for ion and electron transport. Two-dimensional NiMoO_4_ nanosheets possess numerous active sites and multi-access ion paths. Benefiting from above-mentioned advantages, the CuCo_2_O_4_/NiMoO_4_ heterostructures exhibit superior pseudocapacitive performance with a high specific capacitance of 2350 F/g at 1 A/g as well as an excellent cycling stability of 91.5% over 5000 cycles. A solid-state asymmetric supercapacitor based on the CuCo_2_O_4_/NiMoO_4_ electrode as a positive electrode and activated carbon as a negative electrode achieves a high energy density of 51.7 Wh/kg at a power density of 853.7 W/kg. These results indicate that the hybrid micro/nanostructured TMOs will be promising for high-performance supercapacitors.

## 1. Introduction

Supercapacitors, as an emerging energy storage device, have been attracting considerable attention due to their high power density, fast charge/discharge rates and long cycling life [1,2,3,4]. For large-scale practical applications, the energy densities of supercapacitors are still far behind rechargeable batteries [5,6]. In light of the critical parameters that are related to the energy density ($E=1/2CV^{2}$) [7], elevating the specific capacitance is a direct and effective method to increase energy storage. Since the pseudocapacitors rely on rapid faradaic reactions of electrodes to store energy, pseudocapacitive materials usually possess higher capacitance than electric double-layer capacitors [8]. In recent years, ternary transition metal oxides such as NiCo_2_O_4_ [9,10], ZnCo_2_O_4_ [11,12], FeCo_2_O_4_ [13,14] and MnCo_2_O_4_ [15,16] have been extensively studied for the pseudocapacitor applications because they can provide multiple oxidation states for efficient redox reactions. Among various ternary transition metal oxides, CuCo_2_O_4_ is considered a potential material for supercapacitors due to electrochemical activity [17]. Nevertheless, owing to relatively low specific capacitance and electrical conductivity, single-component CuCo_2_O_4_ as an electrode material is not adequate to meet the requirements of a supercapacitor’s application completely. Nowadays, much research effort has been focused on multicomponent composite, such as CuCo_2_O_4_@MnMoO_4_ [18], CuCo_2_O_4_@MoNi-LDH [19] and CuCo_2_O_4_@Co(OH)_2_ [20]. Indeed, these composites can significantly improve electrochemical performance according to data of the above-mentioned research articles. Thus, designing composite materials based on CuCo_2_O_4_ is an effective strategy to achieve high specific capacitance of supercapacitors. NiMoO_4_, as a member of pseudocapacitive materials, possesses a high specific capacitance because of the high electrochemical activity of Ni atoms with different oxidation states and good electrical conductivity of Mo atoms [21]. Therefore, constructing CuCo_2_O_4_@NiMoO_4_ nanostructure composite is expected to achieve high capacitance by combining the advantages of two-component materials. However, poor cycle stability and inferior structural stability of NiMoO_4_-based electrodes resulting from the excessive volume change and aggregation after long-term cycles limit its applications [22,23].

Structural engineering is another critical factor in reaching optimal supercapacitor performance, especially for cycling and rate performances. Among various structures, 2D structures have demonstrated their merits in various applications owing to their anisotropic structure and high surface-to-bulk ratio, which grant a short diffusion path for electrons and ions and rich active sites [24]. For instance, Zhao et al. reported that NiMoO_4_ nanosheets-coated NiCo_2_O_4_ exhibited an ultrahigh specific capacitance of 2806 F/g at 5 A/g and good rate capability with 1408 F/g at 30 A/g [25]. Nevertheless, 2D structures face the problems of self-aggregation and structure collapses during cycling, which lead to irreversible capacitance loss and lifetime decay. Micro/nanostructures with subtle morphologies have been demonstrated to stabilize the skeleton structure during the charge/discharge process. Ravi et al. reported that asymmetric supercapacitors assembled with micro-nano MnCO_3_ showed superior capacitance retention of 98.79% after 10,000 cycles [26]. To further improve the specific capacitance of CuCo_2_O_4_@NiMoO_4_ composites without compromising structural stability, novel micro/nanostructured materials with 2D structure need to be explored.

Herein, we have proposed and validated the rational design and fabrication of the 3D hierarchical CuCo_2_O_4_/NiMoO_4_ heterostructures on Ni foam through a facile and stepwise hydrothermal approach. The combination of 2D microflake CuCo_2_O_4_ assembled 3D architecture and secondary inserted units of NiMoO_4_ ultrathin nanosheets make full use of the merits of individual components and micro/nanostructure. The microflakes assembled island-like CuCo_2_O_4_ serve as the backbone material, and subsequent embedded NiMoO_4_ nanosheets can provide more active sites and electron/ion transport channels to facilitate the reaction kinetics. In addition, building a binder-free integrated structure with current collectors creates a highly efficient electron conducting pathway and avoids a “dead surface”. Therefore, the micro/nanostructure with multicomponent composite electrode exhibits excellent electrochemical performance.

## 2. Results and Discussion

### 2.1. Characterization

**Figure 1:**
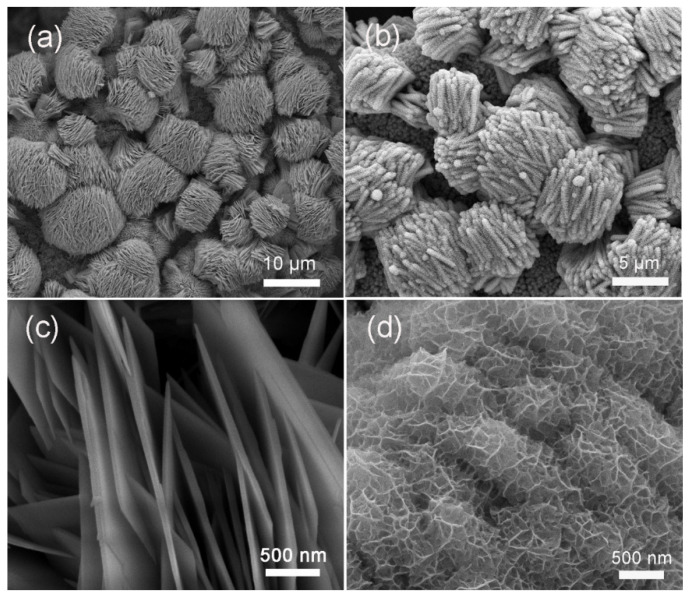
SEM images of CuCo_2_O_4_ island and microflakes (**a**,**c**), CuCo_2_O_4_/NiMoO_4_ micro/nano-heterostructures (**b**,**d**).

Figure 1 displays the morphologies of pristine CuCo_2_O_4_ and CuCo_2_O_4_/NiMoO_4_ heterostructures. As can be seen in Figure 1a, the island-like CuCo_2_O_4_ microstructures are nearly uniformly anchored on Ni foam with an average diameter of about 10 µm. With a closer view (Figure 1c), these islands are assembled by microflakes with a thickness of about 40 nm. Moreover, the microflakes are shown to have plenty of open spaces between these adequately separated microflakes, which is beneficial for electrolyte transportation. The 3D structure can also serve as the ideal conductive skeleton for the subsequent NiMoO_4_ nanosheet growth. From Figure 1b, it can be clearly seen that the interstices between CuCo_2_O_4_ microflakes are filled with ultrathin NiMoO_4_ nanosheet. Under a lower magnification (Figure S1b), the surface of the Ni foam substrate is uniformly covered by the island-like structure while the surface of pristine Ni foam is smooth (Figure S1a), suggesting that CuCo_2_O_4_/NiMoO_4_ anchored on the Ni Foam can be used directly as binder-free integrated electrodes for supercapacitors. The magnified image in Figure 1d shows that the NiMoO_4_ nanosheets are typically interconnected with each other with a thickness of less than 5 nm. Such configuration is of great importance to facilitate electron and ion transfer, increase active sites and maintain structural stability. As a control sample (Figure S2), well-defined NiMoO_4_ nanosheets are nearly uniformly aligned on the Ni foam.

**Figure 2:**
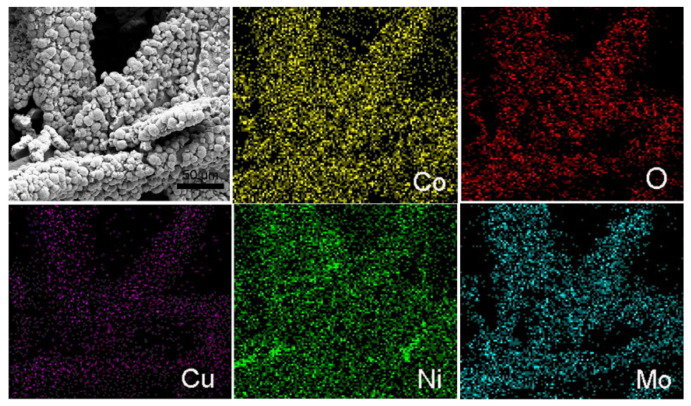
EDS mapping image of CuCo_2_O_4_/NiMoO_4_ micro/nano-heterostructures.

To further identify the structure and composition of the CuCo_2_O_4_ and CuCo_2_O_4_/NiMoO_4_ heterostructures, the elemental mapping is carried out under X-ray spectroscopy (EDS) mapping analysis. The elemental distribution of the CuCo_2_O_4_ microflake structure displays that the elements of Cu, Co and O were distributed uniformly on the whole area (Figure S3). In addition, the Cu/Co atomic ratio is about 0.5 (Figure S4), which matches the formula of CuCo_2_O_4_. After loading the NiMoO_4_ nanosheets, all the elements (Cu, Co, Ni, Mo and O) are detected and homogenously distributed on CuCo_2_O_4_/NiMoO_4_ hybrid heterostructures (Figure 2).

**Figure 3:**
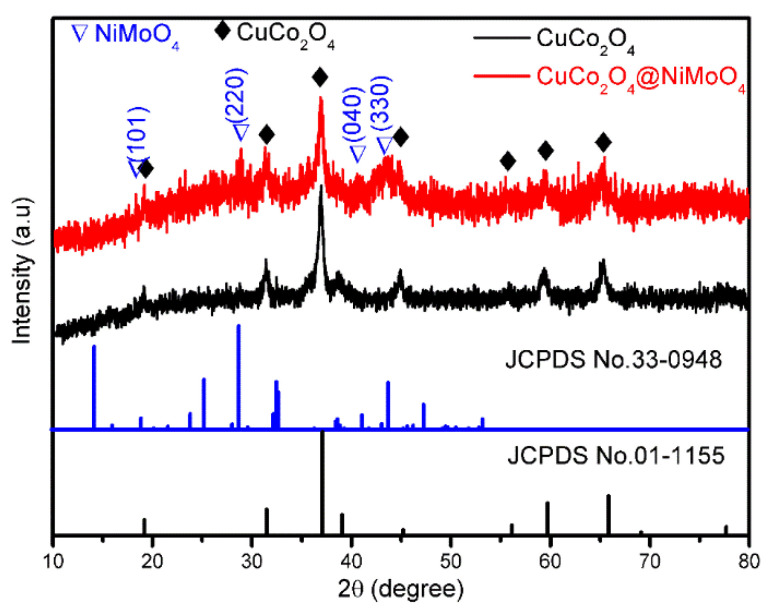
XRD patterns of the CuCo_2_O_4_ microflakes and CuCo_2_O_4_/NiMoO_4_ micro/nano-heterostructures.

Further insights into the crystal structure of CuCo_2_O_4_ and CuCo_2_O_4_/NiMoO_4_ heterostructures are elucidated by XRD patterns. As shown in Figure 3, the obtained patterns of CuCo_2_O_4_ microflakes match well with the standard patterns for the cubic spinel phase of CuCo_2_O_4_ (JCPDS No. 01-1155). For the heterostructures, several weak diffraction peaks attributed to NiMoO_4_ (marked as triangle) are observed except for the peaks of the CuCo_2_O_4_ skeleton (marked as diamond), indicating relatively low crystallinity compared with the CuCo_2_O_4_ phase. Moreover, Figure S5 shows the identified peaks of the control sample, which can be mainly assigned to monoclinic NiMoO_4_ (JCPDS No. 45-0142) with a small amount of α-NiMoO_4_ phase (JCPDS No. 33-0948).

**Figure 4:**
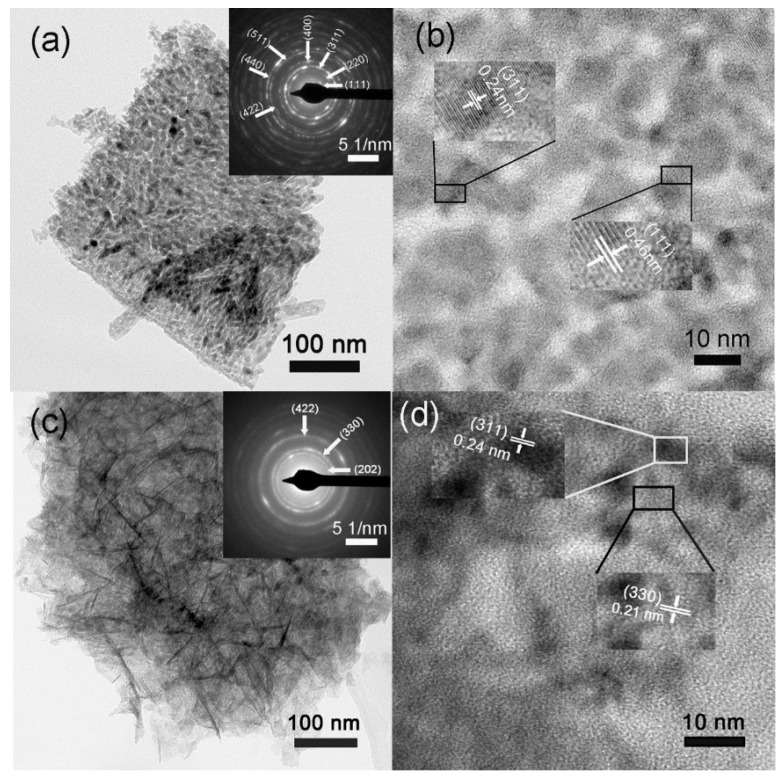
(**a**) TEM image of CuCo_2_O_4_ nanosheets and corresponding SAED pattern, (**b**) HRTEM image of the CuCo_2_O_4_ nanosheets, (**c**) TEM image of CuCo_2_O_4_/NiMoO_4_ heterostructures and corresponding SAED pattern and (**d**) HRTEM image of the CuCo_2_O_4_/NiMoO_4_ heterostructures.

The detailed structural characterization of CuCo_2_O_4_ and CuCo_2_O_4_/NiMoO_4_ are further investigated by TEM and HRTEM. Under a low-magnification TEM image (Figure S6a), these CuCo_2_O_4_ microflakes are shown to have a smooth surface. An enlarged TEM view (Figure 4a) reveals that the microflakes are porous and composed of many interconnected crystalline nanoparticles (~10 nm). Furthermore, the selected-area electron diffraction (SAED) patterns in the inset of Figure 4a demonstrate the polycrystalline nature of the CuCo_2_O_4_ skeleton, which is consistent with the XRD result. HRTEM measurements (Figure 4b) also clearly display two sets of visible lattice fringes with an interplanar spacing of 0.46 nm and 0.24 nm, corresponding to the (111) and (311) planes of CuCo_2_O_4_. Compared with the smooth CuCo_2_O_4_ microflakes, noticeable wrinkle-like structures are found on the surface of heterostructures (Figure 4c, Figure S6b). Meanwhile, the well-defined SAED pattern is also observed in the inset of Figure 4c, and the three diffraction rings correspond to the (422), (330) and (202) crystal planes of NiMoO_4_. Figure 4d shows the HRTEM image of hybrid heterostructures. The measured lattice spacing of 0.21 nm and 0.24 nm are in good agreement with the (330) plane of NiMoO_4_ and (311) plane of CuCo_2_O_4_, respectively. The above results demonstrate the formation of CuCo_2_O_4_/NiMoO_4_ heterostructures.

### 2.2. Electrochemical Measurements

**Figure 5:**
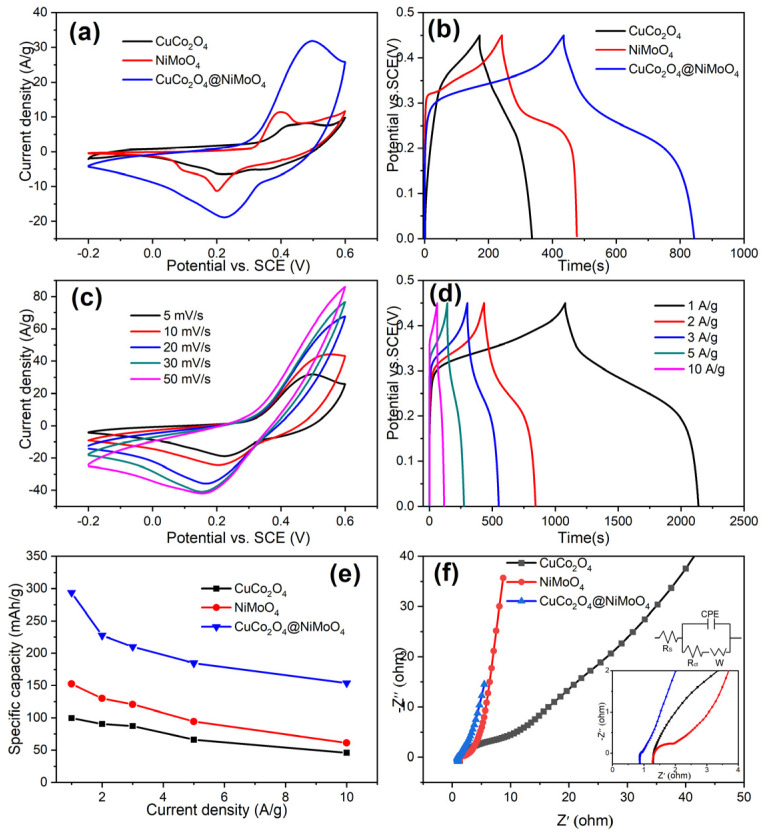
(**a**) CV curves of the CuCo_2_O_4_, NiMoO_4_ and CuCo_2_O_4_/NiMoO_4_ electrodes at 5 mV/s, (**b**) GCD curves of these electrodes at 2 A/g, (**c**) CV curves of the CuCo_2_O_4_/NiMoO_4_ electrode at various scan rates, (**d**) GCD curves of the CuCo_2_O_4_/NiMoO_4_ electrode at different current densities, (**e**) calculated specific capacitance of the three electrodes as a function of current density and (**f**) EIS Nyquist plots, inset showing the enlarged picture of high-frequency region and electrical equivalent circuit.

To evaluate CuCo_2_O_4_@NiMoO_4_ heterostructures as a potential electrode material for the supercapacitor, CV and GCD tests are performed to measure electrochemical performance in a 6 M KOH solution in a three-electrode system. Figure 5a shows the CV curves of the pure NiMoO_4_, CuCo_2_O_4_ and CuCo_2_O_4_/NiMoO_4_ heterostructures at the scan rate of 5 mV/s. It should be noted that the enclosed CV curve area of the CuCo_2_O_4_/NiMoO_4_ heterostructures is apparently larger than those of the pure NiMoO_4_ or CuCo_2_O_4_, indicating that the CuCo_2_O_4_/NiMoO_4_ heterostructures have a larger specific capacitance than the other two materials. This fact can be further confirmed by GCD curves. As shown in Figure 5b, CuCo_2_O_4_/NiMoO_4_ heterostructures display a much longer discharge time in comparison with the other two materials at a current density of 2 A/g. The results prove that the NiMoO_4_ nanosheets embedded in island-like CuCo_2_O_4_ skeleton can significantly improve the capacitive performance. Figure 5c shows CV curves of CuCo_2_O_4_/NiMoO_4_ at different scan rates. All CV curves have similar shapes within a pair of redox peaks. The same phenomenon is also observed in a single component of either CuCo_2_O_4_ skeleton (Figure S7a) or NiMoO_4_ nanosheets (Figure S7c). Apparently, individual components (CuCo_2_O_4_ or NiMoO_4_) and composite both show pseudocapacitive behavior due to faradaic redox reactions [27]. Moreover, with the increasing of scan rates from 5 mV/s to 50 mV/s, the oxidation peak shifts towards a more positive position and the reduction peak towards a more negative position, which is supposed to be related to the internal resistance of the electrode and limitation of charge transfer kinetics [28]. It is worth noting that smaller shifts in the peak position of the CuCo_2_O_4_/NiMoO_4_ electrode corresponds to that of CuCo_2_O_4_ or NiMoO_4_ electrode in a tenfold increase in the scan rate, implying relatively low resistance and fast redox reactions. GCD curves further intuitively explain the phenomenon. As can be seen in Figure 5d, good symmetry of the profiles reveals the excellent reversibility of the charge/discharge behavior. A pair of potential plateaus in the charge and discharge processes is observed, which is consistent with the above CV results. The GCD curves of CuCo_2_O_4_ and NiMoO_4_ electrodes at various current densities are shown in Figure S7b,d, respectively. The calculated specific capacitance as a function of the discharge current density is plotted in Figure 5e. Specifically, CuCo_2_O_4_/NiMoO_4_ electrode delivers an ultrahigh capacitance of 2350 F/g at a current density of 1 A/g and an impressive capacitance as high as 1235 F/g can be achieved even at a current density of 10 A/g. In total, 52.5% of the initial capacitance is retained when the current density increases from 1.0 to 10 A/g, indicating excellent high-rate capability. Compared with CuCo_2_O_4_/NiMoO_4_ electrodes, CuCo_2_O_4_ and NiMoO_4_ electrodes show inferior performance in terms of specific capacitance and rate capability (Table 1). EIS techniques are used to investigate the insights into the advantages of these electrodes. In Figure 5f, all Nyquist plots consist of three regions according to the different frequency ranges corresponding to the different interfacial processes. Briefly, the high-frequency intercept on the real Z′ axis represents the series resistance (Rs). In the middle-frequency region, the semicircle can be attributed to the charge transfer resistance (Rct) on the electrode surface [29]. As shown in the inset of Figure 5f, the CuCo_2_O_4_/NiMoO_4_ electrode is found to have a much smaller intercept and diameter of the semicircle than the CuCo_2_O_4_ or NiMoO_4_ electrode, indicating a lower Rs and Rct of the CuCo_2_O_4_/NiMoO_4_ electrode. The straight line in the low frequency is associated with the ion diffusion of electrolytes. The almost vertical straight lines at a low frequency of both the CuCo_2_O_4_/NiMoO_4_ electrode and the NiMoO_4_ electrode demonstrated their superior electrolyte ionic diffusion behavior. The fitted values can provide a more intuitive interpretation, as shown in Table S1. The R_s_ value is rather small and similar for three electrodes, showing the advantage for in situ growth. The CuCo_2_O_4_/NiMoO_4_ and NiMoO_4_ electrode have similar values of R_ct_ and W, which are lower than that of the CuCo_2_O_4_ electrode. The rapid charge transfer rate and ion diffusion of CuCo_2_O_4_/NiMoO_4_ is attributed to thin nanosheets and the in situ growth of CuCo_2_O_4_ on Ni Foam. Therefore, the improved conductivity and mass transport in CuCo_2_O_4_/NiMoO_4_ micro/nanostructure enhances its electrochemical performance.

**Figure 6:**
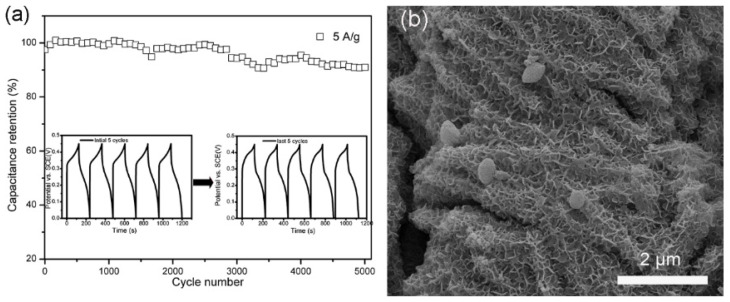
(**a**) Cycling performance of CuCo_2_O_4_/NiMoO_4_ electrode at 5 A/g. (inset: GCD curves of the first 5 cycles and the last 5 cycles). (**b**) SEM image of CuCo_2_O_4_/NiMoO_4_ electrode after 5000 cycles.

Furthermore, cycle stability is evaluated by repeating a charge/discharge process of the electrode materials. As the skeleton, island-like CuCo_2_O_4_ structure manifests exceptional cycling stability and is able to reach 91.0% of its initial value even after 5000 cycles (Figure S8a). After inserting NiMoO_4_ nanosheets into the skeleton, the CuCo_2_O_4_/NiMoO_4_ still maintains similar or even better cycling stability (Figure 6a). On the other hand, NiMoO4 nanosheets show a capacitance loss of 30.3% after only 3000 cycles (Figure S8c). Therefore, the unique construction enhances structural stability: NiMoO_4_ nanosheets are inserted into layered CuCo_2_O_4_ microflakes, which inhibit the microflake collapse and self-agglomeration of nanosheets, while CuCo_2_O_4_ microflakes possess robust stability first. As indicated by the FESEM images of electrode materials after the cycling test (Figure 6b and Figure S8b), the structure of both island-like CuCo_2_O_4_ and CuCo_2_O_4_/NiMoO_4_ are almost preserved, whereas NiMoO_4_ nanosheets suffer from severe aggregation and deformation after long-term cycles (Figure S8d). Thus, it can be further inferred that enhanced cycling stability of CuCo_2_O_4_/NiMoO_4_ heterostructures results from the unique structural features owing to the intrinsic rigid island-like skeleton and mutual support between nanosheets and microflakes. The comparison of the electrochemical performance of the three electrode materials is listed in Table 1. The electrochemical performance of CuCo_2_O_4_@NiMoO_4_ heterostructures not only exceeds that of either CuCo_2_O_4_ island-like structure or NiMoO_4_ nanosheets but also is superior to that of many previously reported mixed-metal oxides in terms of specific capacitance and cycling performance (Table S2).

**Figure 7:**
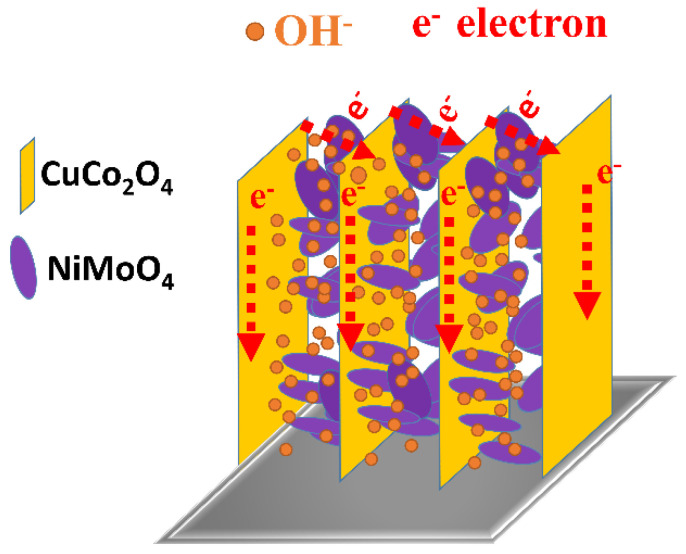
Schematic illustration of the electron transport pathway and ion diffusion of the CuCo_2_O_4_/NiMoO_4_ on Ni foam.

Such desirable pseudocapacitive performance of the CuCo_2_O_4_/NiMoO_4_ heterostructures can be explained as electron and ion transfer of unique structures. As illustrated in Figure 7, the electron/ion transfer path is as follows: (1) Two-dimensional NiMoO_4_ nanosheets connect with each other to form the horizontal transport channel of electron and ion. (2) Two-dimensional CuCo_2_O_4_ microflakes are vertically grown on Ni foam, which are favorable for fast ion diffusion and electron transfer reaction. (3) Porous structures on microflakes and between nanosheets can increase specific surface area and facilitate electrolyte penetration. As shown in Figure S9, the BET-specific surface areas of heterostructures, CuCo_2_O_4_ microflakes and NiMoO_4_ nanosheets are 98.5, 38.3 and 31.1 m^2^/g, respectively. From pore size distribution analysis, heterostructures composite has richer pore structure than the other two materials. Similarly, the micro/nanostructure is used to provide insights into superior cycling lifetime: (1) Microflakes assembled a 3D island-like structure provide good mechanical stability, and ensure structural integrity under continuous charge/discharge cycles. (2) Ultrathin NiMoO_4_ nanosheets as secondary inserted units embedded inside CuCo_2_O_4_ framework, which have mutually reinforcing effects and provide more active sites. According to the above analysis, a hierarchical micro/nanostructure achieves the synergistic effect between CuCo_2_O_4_ microflakes and NiMoO_4_ nanosheets by combining the advantages of both composition and unique structure. electrochemical performances of composite materials with micro/nanostructure surpass over that of single-component oxides or a 2D single structure.

**Figure 8:**
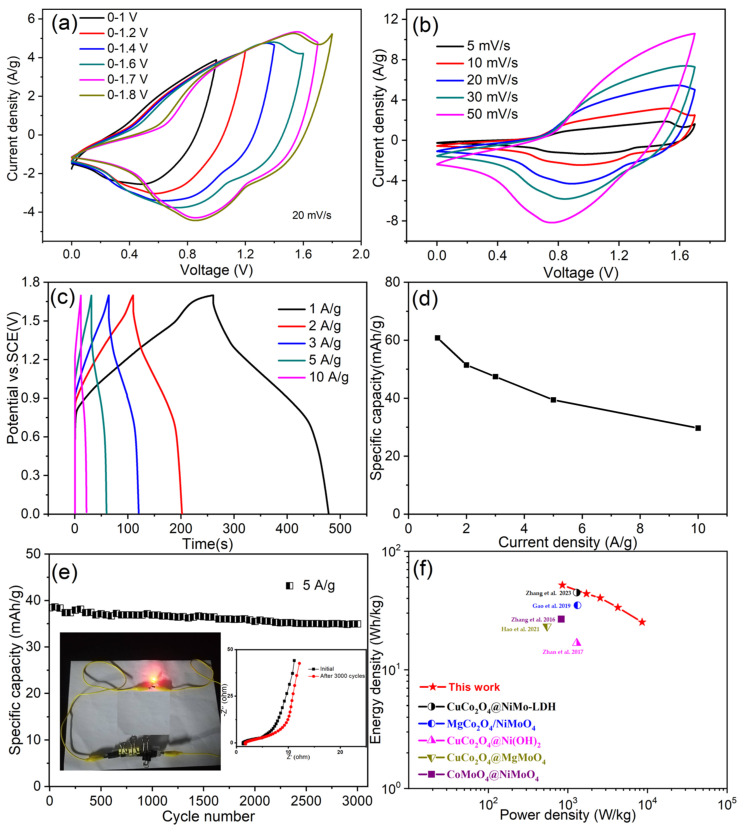
(**a**) CV curves of the device with different potential windows varying from 1.0 to 1.8 V at 20 mV/s. (**b**) CV curves of the device at various scan rates. (**c**) GCD curves of the device at different current densities. (**d**) Calculated specific capacitance of the device as function of current density. (**e**) Cyclic stability of the device at 5 A/g. (Inset: the red LED powered by the device and Nyquist plots of solid-state supercapacitor before and after 3000 cycles). (**f**) Ragone plots of the device compared with previously reported data [19,33,34,35,36].

In order to evaluate the practical application, an electrochemical asymmetric supercapacitor (ASC) is assembled by using the as-prepared CuCo_2_O_4_/NiMoO_4_ heterostructures as a positive and activated carbon as a negative with PVA/KOH gel as the electrolyte. By matching the charges stored in the two electrodes according to formula: $m^{+}/m^{-}=(C^{-}\times \Delta V^{-})/(C^{+}\times \Delta V^{+})$, the optimal mass ratio between AC and CuCo_2_O_4_@NiMoO_4_ is calculated to be around 7.74:1 based on the obtained CV results and GCD (Figure S10a–c). Figure 8a shows CV curves of the device collected at 20 mV/s with different potential windows ranging from 1.0 to 1.8 V. The result indicates that the maximum potential of the prepared device is 1.7 V because an obvious oxygen evolution occurs when the operating potential window exceeds 1.7 V. After obtaining this critical parameter, CV tests are researched at various scans with a fixed potential of 1.7 V (Figure 8b). All the CV shapes remained with barely any deformations with the increasing sweeping rate, demonstrating desirable charge/discharge behavior with superior reversibility. The GCD curves with different current densities from 1 to 10 A/g are shown in Figure 8c. The specific capacitance of the device as a function of current density is presented in Figure 8d. A remarkably high specific capacitance of 128.8 F/g is obtained at 1 A/g. Even at a high current density of 10 A/g, the device still has a specific capacitance of 63.2 F/g, which retained about 49.1% of the initial capacitance with a tenfold increase in the current density. Figure 8e shows the cycling stability of the device at a current of 5 A/g. After 3000 charge/discharge cycles, only about 9.2% capacitance loss was observed, revealing its excellent cycling stability. To find out the reason for capacity decay, nyquist plots are investigated (inset of Figure 8e). It can be clearly seen that intercept and semicircle at high-frequency region become slightly larger after cycles while slopes at a low frequency remain almost unchanged. The result confirms increased charge transfer resistance and internal resistance, which is associated with lifetime decay. The device can power a red commercial LED light (2.0 V) using two electrodes in series, as shown in Figure 8e (inset). Furthermore, the device delivers a high energy density of 51.7 Wh /kg at a power density of 853.7 W /kg and an energy density of 25.2 Wh /kg even at a high power density of 8558.2 W /kg, which is superior to the recently reported value (Figure 8f) [30,31,32,33].

## 3. Experimental

All of the reagents used in the experiments were of analytical grade and were used without further purification. Prior to the synthesis, the Ni foam was cleaned by acetone, ethanol and deionized water for 20 min in sequence with sonication. CuCo_2_O_4_, NiMoO_4_ and CuCo_2_O_4_/NiMoO_4_ were prepared by the hydrothermal method. In this work, activated carbon (YP50) was purchased from Kuraray Co., Ltd. (Tokyo, Japan). The other reagents were purchased from Sinopharm Chemical Reagents Co., Ltd. (Shanghai, China).

### 3.1. Synthesis of CuCo_2_O_4_ Island-like Structure

The materials were synthesized similarly according to previously reported procedures with minor modifications [34]. In brief, Co(NO_3_)_2_·3H_2_O (2.0 mmol), Cu(NO_3_)_2_·3H_2_O (1.0 mmol) and urea (5 mmol) were dissolved in 40 mL deionized water and ethanol (volume ratio = 5:3). The as-obtained solution and nickel foam were transferred to a 50 mL Teflon-lined stainless-steel autoclave. Subsequently, the reaction mixture was maintained at 120 °C for 6 h in an electric oven. After the reaction, the solution was cooled down to room temperature naturally. The as-obtained sample was rinsed with distilled water and ethanol thoroughly and dried in an oven at 60 °C for 12 h. Finally, the sample was annealed at 350 °C for 3 h, and the CuCo_2_O_4_ microflake’s island-like structure was obtained.

### 3.2. Synthesis of CuCo_2_O_4_/NiMoO_4_ Heterostructures on Ni Foam

**Figure 9:**
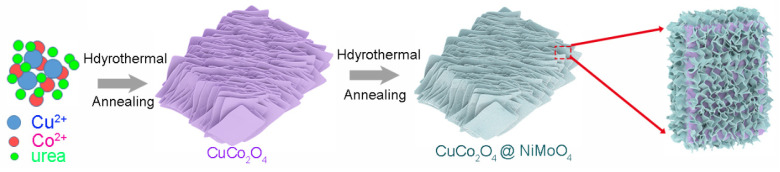
Schematic illustration for the fabrication of CuCo_2_O_4_/NiMoO_4_ micro/nano-heterostructures (purple and light blue represent CuCo_2_O_4_ microflakes and NiMoO_4_ nanosheets, respectively).

The synthesized CuCo_2_O_4_ was used as the skeleton for the growth of NiMoO_4_ active materials. In total, 0.249 g Ni(CH_3_COO)_2_·4H_2_O, 0.2 g (NH_4_)_6_Mo_7_O_24_·4H_2_O, and 0.24 g urea were dissolved into 40 mL DI water. The mixed solution and CuCo_2_O_4_@Ni foam were transferred to a 50 mL Teflon-lined stainless-steel autoclave. The autoclave was sealed and maintained at 140 °C for 2 h and then cooled to room temperature naturally. The sample was taken out and rinsed with distilled water and alcohol several times. Finally, the CuCo_2_O_4_/NiMoO4 was obtained by annealing at 400 °C for 2 h in the argon atmosphere. As shown in Figure 9, NiMoO_4_ nanosheets (light blue) were tightly anchored on the surface of CuCo_2_O_4_ microflakes (purple) to form micro/nano-heterostructure. As a control experiment, NiMoO_4_ nanosheets were synthesized according to a reported hydrothermal method [35]. Ni(NO_3_)_2_ 6H_2_O (1 mmol) and Na_2_MoO_4_·2H_2_O (1 mmol) were dissolved in a mixed solvent of 15 mL of H_2_O and 15 mL of ethanol. The mixed solution and cleaned Ni Foam were transferred to the autoclave. Afterwards, the autoclave was sealed and maintained at 160 °C for 6 h to synthesize Ni-Mo precursor nanosheet arrays. The final product was obtained by annealing at 450 °C for 2 h in the argon atmosphere.

### 3.3. Materials Characterization

The structure and phase were characterized by X-ray diffraction (XRD, Ultima IV, Rigaku, Tokyo, Japan) using Cu/Kα radiation (λ = 0.154 nm). Morphologies were observed by a field-emission scanning electron microscope (FESEM, Inspect F50, FEI, Hillsboro, OR, USA). The surface elemental analysis was confirmed by the elemental mapping using X-ray energy dispersive spectroscopy (EDS). More structural information was detected by transmission electron microscopy (TEM) and high-resolution transmission electron microscope (HRTEM, G2 20, Tecnai, Hillsboro, OR, USA). Selected area electron diffraction (SAED) patterns were recorded by a Gatan CCD camera in a digital format.

### 3.4. Electrochemical Measurements

The electrochemical tests including cyclic voltammetry (CV), galvanostatic charge/discharge (GCD) measurement and electrochemical impedance spectra (EIS) were performed on CHI 760E electrochemical workstation using three-electrode systems in 3.0M KOH solution with Pt foil as the counter electrode and saturated calomel electrode (SCE) as the reference electrode. EIS was carried out in the frequency range of 0.01 Hz~1 MHz at the open circuit potential with an AC potential amplitude of 5 mV. The EIS test data was fitted with Zview software (Scribner Associates, version 2.9c). Cycling performance was conducted using a LAND battery program-control test system (Wuhan LAND Electronics, Wuhan, China) in the potential range of 0~0.45 V. The corresponding mass loadings of CuCo_2_O_4_, CuCo_2_O_4_/NiMoO_4_ and NiMoO_4_ were approximately 1.6, 2.7 and 2.1 mg cm^−2^, respectively.

### 3.5. Assemble of Asymmetric Supercapacitor

A solid-state asymmetric supercapacitor assembled by CuCo_2_O_4_/NiMoO_4_ as a positive electrode, activated carbon (AC) as a negative electrode, and polyvinyl alcohol (PVA)/KOH gel both as the electrolyte and separator were carried out. The AC electrode was prepared by mixing 95 wt% of activated carbon and 5 wt% of polytetrafluoroethylene and then spread on a 10 mm × 20 mm Ni foam. (PVA)/KOH gel was synthetized by the following procedure [36]: 6 g of KOH was dissolved in 60 mL of deionized water, followed by the addition of 6 g of PVA power. The mixture was heated to 90 °C under stirring until the solution became clear. The PVA/KOH polymer electrolyte was obtained at room temperature. The as-prepared CuCo_2_O_4_/NiMoO_4_/Ni and AC/Ni were soaked in PVA/KOH gel for 10 min under ultrasonic treatment to allow the electrolyte to diffuse into nanoporous active materials. The two electrodes were fabricated into a sandwich-structure solid-state supercapacitor when the PVA-KOH gel electrolyte was solidified under hot air.

## 4. Conclusions

In summary, we have successfully developed a hierarchical CuCo_2_O_4_/NiMoO_4_ heterostructures by using microflakes assembled island-like CuCo_2_O_4_ as a skeleton and ultrathin nanosheets NiMoO_4_ as a secondary structure. Owing to the combination of the chemical composition and the spatial scale/dimension, such a novel electrode material can effectively provide a large amount of reactive active sites, multi-access channels of ions and the favorable structural stability. The resulting CuCo_2_O_4_/NiMoO_4_ heterostructures exhibited greatly enhanced electrochemical performance. The asymmetric supercapacitor achieves a high energy density of 51.7 Wh/kg at a power density of 853.7 W/kg and a superior cyclability with a capacitance retention of 90.8% after 3000 cycles.

## Figures and Tables

**Table 1:** Comparison of the three-electrode performance based on similar composition and morphology.

Electrode Materials	Max Capacitance	Min Capacitance	Rate Capability	Retention (Cycles)
NiMoO_4_ nanosheet arrays	1220 (1 A/g)	490 (10 A/g)	40.2%	69.7% (3000)
CuCo_2_O_4_ nanosheet-assembled island-like	797 (1 A/g)	366 (10 A/g)	45.9%	91.0% (5000)
CuCo_2_O_4_/NiMoO_4_ hierarchically structure	2350 (1 A/g)	1235 (10 A/g)	52.5%	91.5% (5000)

## Data Availability

Not applicable.

## Supplementary Materials

The following supporting information can be downloaded at: https://www.mdpi.com/article/10.3390/molecules28196840/s1, Figure S1: SEM images (a) bare Ni foam (b) Ni/CuCo_2_O_4_/NiMoO_4_; Figure S2: SEM image of NiMoO_4_ nanosheet; Figure S3: EDS mapping image of CuCo_2_O_4_ microflakes; Figure S4: EDS spectrum of the elements Co, Cu and O; Figure S5: XRD pattern of NiMoO_4_ nanosheet; Figure S6: TEM images (a) CuCo_2_O_4_ microflakes (b) CuCo_2_O_4_/NiMoO_4_ micro/nano-heterostructures; Figure S7: CV curves of (a) CuCo_2_O_4_ (c) NiMoO_4_ at various scan rates. GCD curves of (b) CuCo_2_O_4_ (d) NiMoO_4_ at different current densities; Figure S8: Cycling performance of (a) CuCo_2_O_4_ electrode (c) NiMoO_4_ electrode at 2 A/g. (inset: GCD curves of the first 5 cycles and the last 5 cycles). SEM images of (c) CuCo_2_O_4_ electrode (d) NiMoO_4_ electrode after 5000 cycles; Figure S9: Nitrogen adsorption–desorption isotherms and pore size distribution (inset) of CuCo_2_O_4_, NiMoO_4_ and CuCo_2_O_4_/NiMoO_4_; Figure S10: CV curve (a) and GCD curve (b) of AC electrode and CV curve of (c) CuCo_2_O_4_/NiMoO_4_ electrode at 10 mV/s; Table S1: The fitted parameters of three electrodes; Table S2: Various pseudocapacitive electrodes in supercapacitors. References [19,22,32,34,37,38,39,40] are cited in the Supplementary Materials.

## Sample Availability

Samples of the Ni/CuCo_2_O_4_, Ni/NiMoO_4_ and Ni/CuCo_2_O_4_/NiMoO_4_ are available from the authors.
