# Supplementary material for: NiMoO4 Nanosheets Embedded in Microflake-Assembled CuCo2O4 Island-like Structure on Ni Foam for High-Performance Asymmetrical Solid-State Supercapacitors
Source: Molecules. 2023 Sep 28;28(19):6840. doi: 10.3390/molecules28196840 (PMC10574438; doi:10.3390/molecules28196840)
Supplement: Supplementary file 1 [file molecules-28-06840-s001.zip › molecules-2598231-supplementary.pdf]

$NiMoO_4$ nanosheets embedded in microflakes-assembled $CuCo_2O_4$ island-like structure on Ni foam for high-performance asymmetrical solid-state supercapacitors

Gaofeng Li [1*], Lingling Chen [2] and Longfei Li [2]

[1] Institute of Advanced Energy Storage Technology and Equipment, Faculty of Mechanical Engineering and Mechanics, Ningbo University, Ningbo, 315211, China;
[2] Institute of Advanced Energy Storage Technology and Equipment, School of Materials Science and Chemcal Engineering, Ningbo University, Ningbo, 315211, China; 664825847@qq.com; 2219926828@qq.com;
* Correspondence: ligaofeng@nbu.edu.cn;

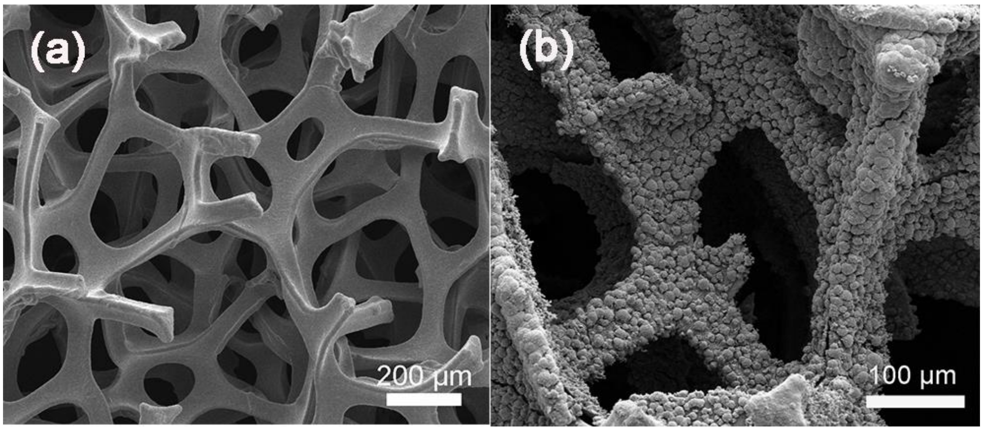

Figure S1. SEM images (a) bare Ni foam (b) Ni/$CuCo_2O_4$/$NiMoO_4$.

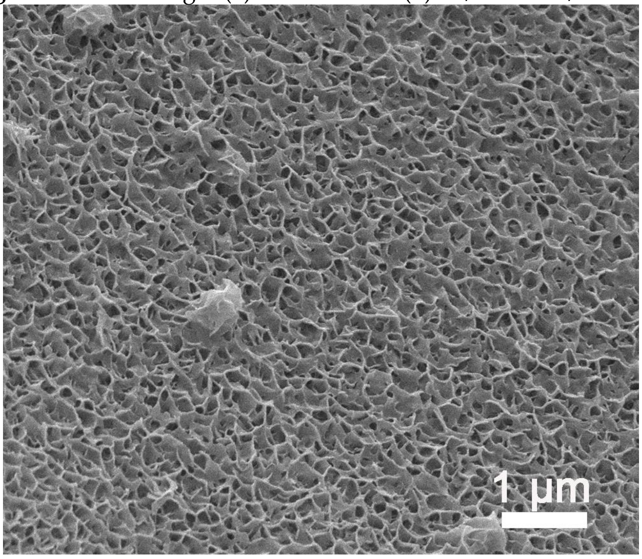

Figure S2. SEM image of $NiMoO_4$ nanosheet.

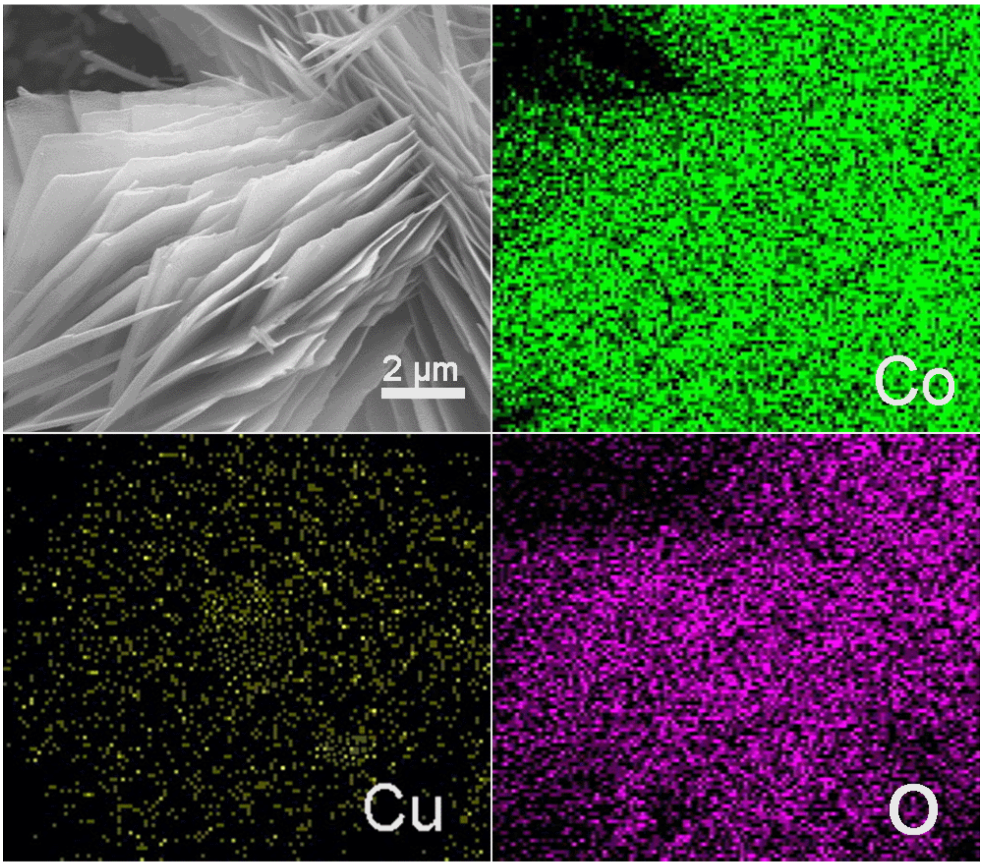

Figure S3. EDS mapping image of $CuCo_2O_4$ microflakes.

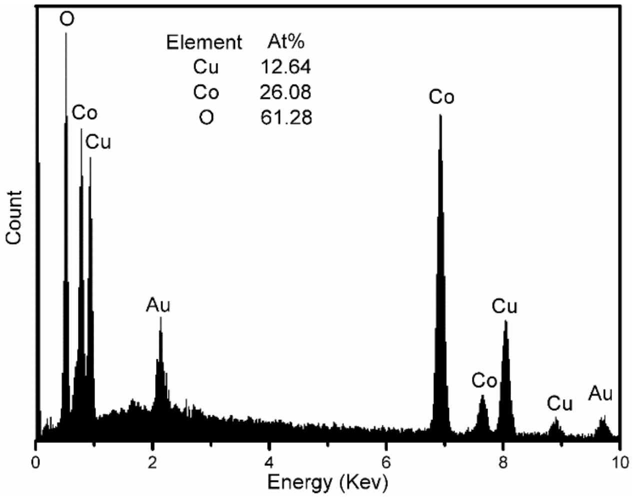

Figure S4. EDS spectrum of the elements Co, Cu and O.

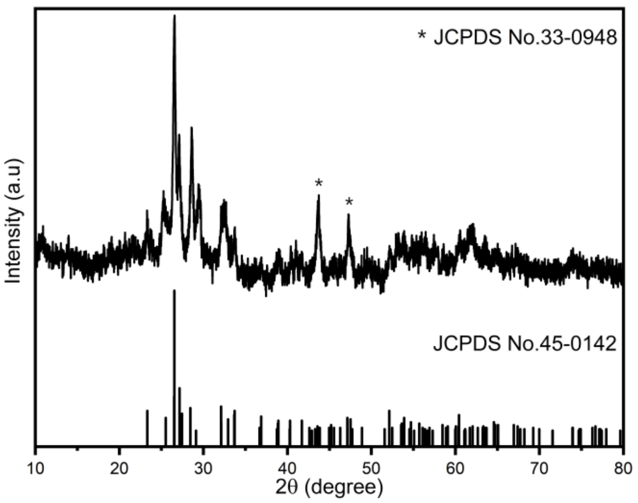

Figure S5. XRD pattern of $NiMoO_4$ nanosheet.

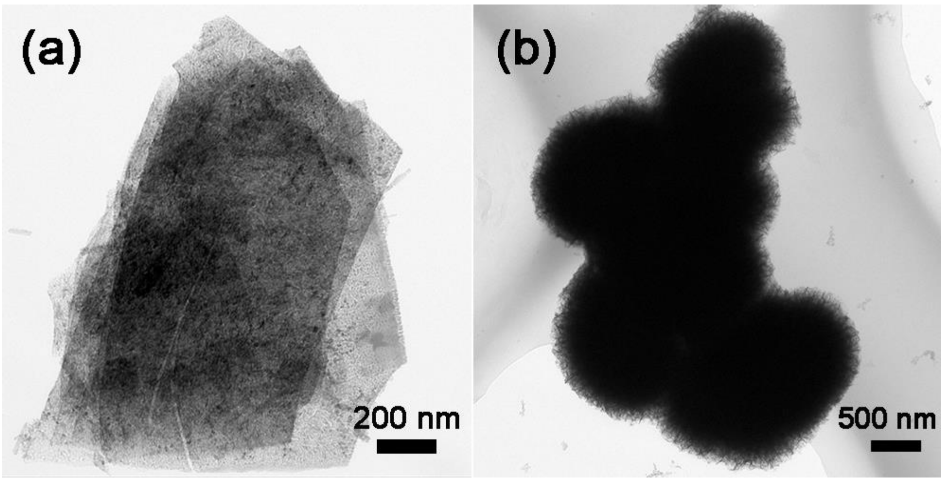

Figure S6. TEM images (a) $CuCo_2O_4$ microflakes (b) $CuCo_2O_4/NiMoO_4$ micro/nano-heterostructures.

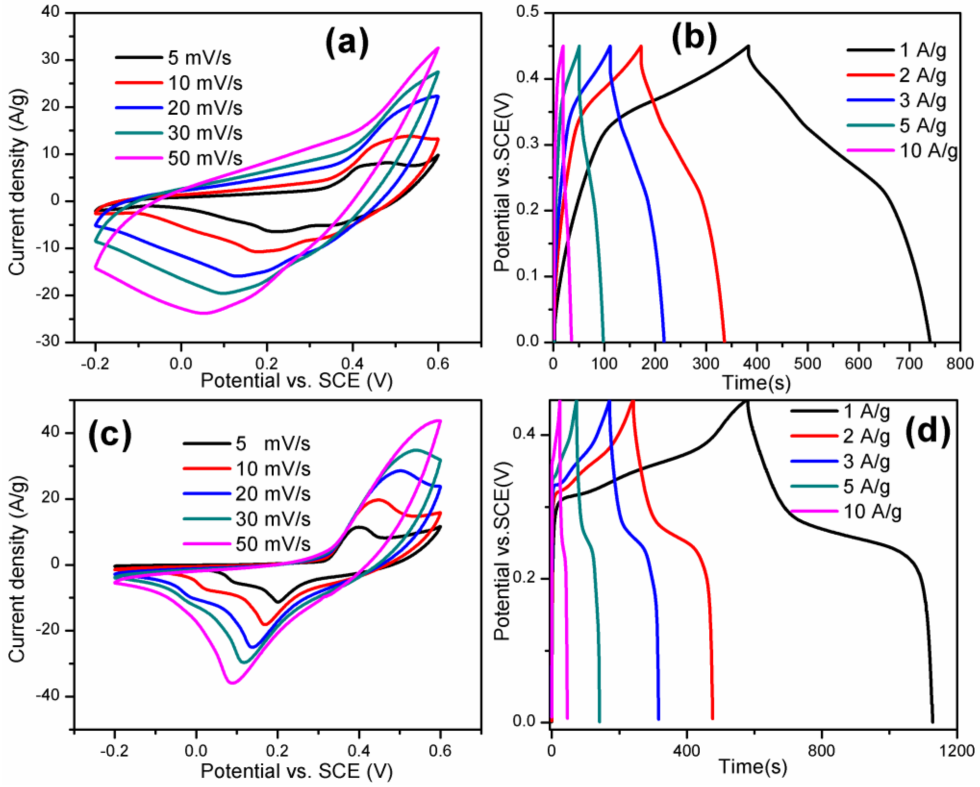

Figure S7. CV curves of (a) $CuCo_2O_4$ (c) $NiMoO_4$ at various scan rates. GCD curves of (b) $CuCo_2O_4$ (d) $NiMoO_4$ at different current densities.

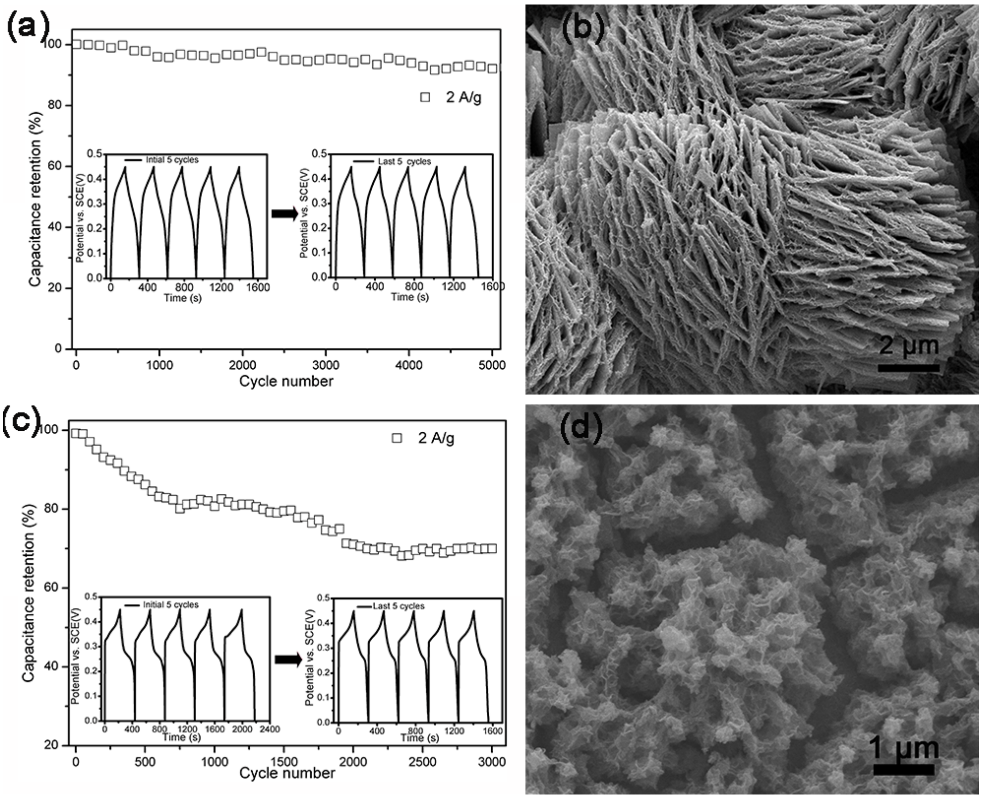

Figure S8. Cycling performance of (a) $CuCo_2O_4$ electrode (c) $NiMoO_4$ electrode at 2 A/g. (inset: GCD curves of the first 5 cycles and the last 5 cycles). SEM images of (b) $CuCo_2O_4$ electrode after 5000 cycles (d) $NiMoO_4$ electrode after 3000 cycles.

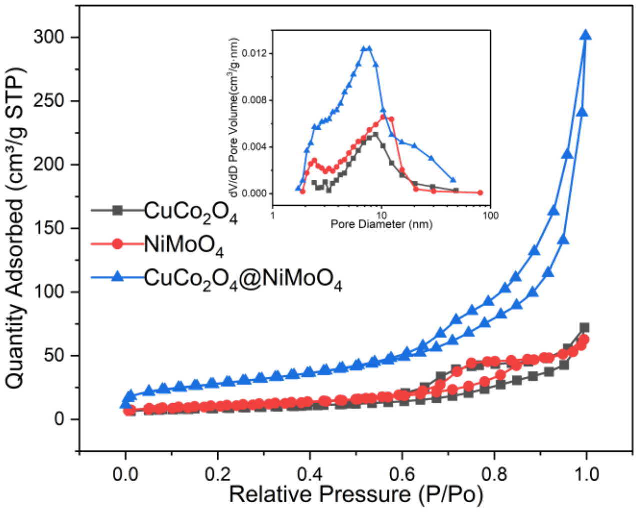

Figure S9. Nitrogen adsorption-desorption isotherms and pore size distribution (inset) of $CuCo_2O_4$, $NiMoO_4$ and $CuCo_2O_4$/$NiMoO_4$ (powder from the Ni foam substrate).

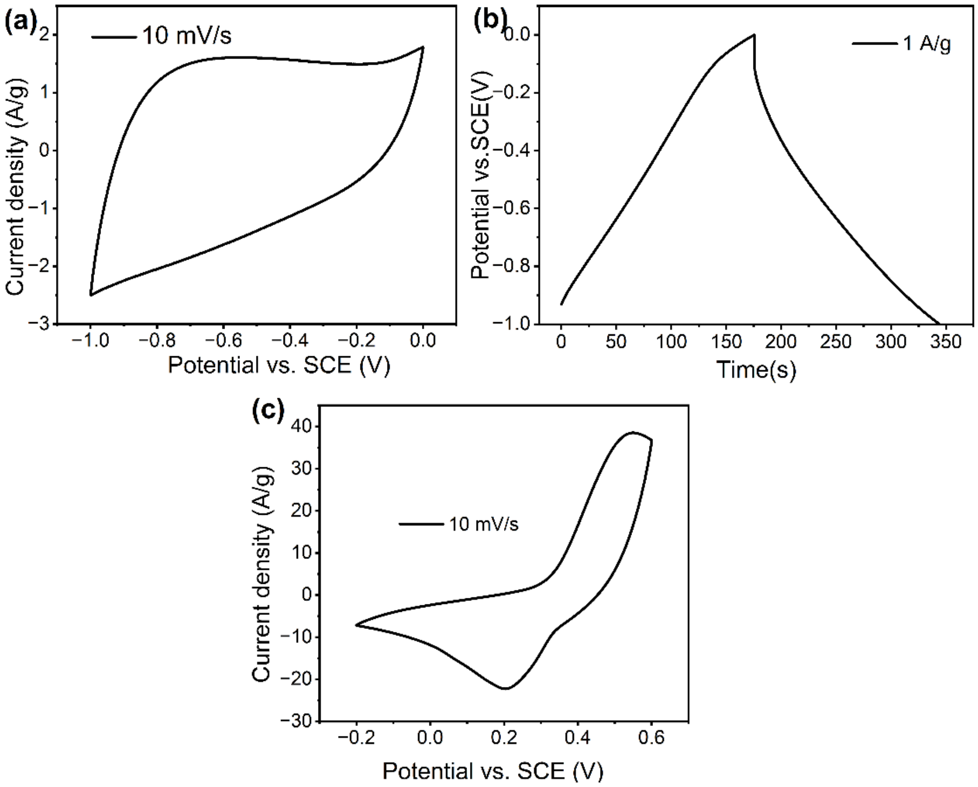

Figure S10. CV (a) and GCD curves (b) of AC electrode. CV curve (c) of $CuCo_2O_4$/$NiMoO_4$ electrode.

Table S1: The fitted parameters of three electrodes.

Sample	R_s (Ω)	R_{ct} (Ω)	W (Ω)
$CuCo_2O_4$ microflakes	1.27	8.27	9.28
$NiMoO_4$ nanosheets	1.36	0.38	5.53
$CuCo_2O_4$/$NiMoO_4$ heterostructures	0.93	1.14	7.54

Table S2: Various pseudocapacitive electrodes in supercapacitors.

Electrode materials	（A/g or mA cm^{-2} ）	Capacitance F g^{-1} (Current density)	Retention (Cycles)	Ref
$NiMoO_4$ wires	1.2 A g^{-1}	1517	76.9 % (4000)	[S1]
$NiMoO_4$/N-doping of graphene	1 A g^{-1}	1913	94.2 % (5000)	[S2]
$NiMoO_4$/rGo composites	1 A g^{-1}	1274	81.1 %(1000)	[S3]
carbon nanofibers embedded with $NiMoO_4$ nanoparticles	1 A g^{-1}	1438	88 %/(3000)	[S4]
$CuCo_2O_4$@MoNi-LDH	1 A g^{-1}	1286	88 %(6000)	[S5]
$CuCo_2O_4$/$MnCo_2O_4$ heterostructures	0.5 A g^{-1}	1434	81.4 %(5000)	[S6]
$CuCo_2O_4$@$MgMoO_4$ composites	1 A g^{-1}	1153	76.6 %(2000)	[S7]
$NiMoO_4$@MoS_2 nanorods	1 A g^{-1}	2246.7	88.4 %(5000)	[S8]
$CuCo_2O_4$/$NiMoO_4$ heterostructures	1 A g^{-1}	2350	91.5 %(5000)	**This work**

References

1. Guo, D.; Luo, Y.; Yu, X.; Li, Q.; Wang, T. High performance $NiMoO_4$ nanowires supported on carbon cloth as advanced electrodes for symmetric supercapacitors. *Nano Energy* **2014**, *8*, 174-182.
2. Feng, X.; Wang, Jing. N, D.; Zhang, J.; Xia, M.; Wang, Y.; Hao, Y. Heterostructure arrays of $NiMoO_4$ nanoflakes on N-doping of graphene for high-performance asymmetric supercapacitors. *J. Alloys Compd.* **2020**, *816*, 152625.
3. Liu, T.; Chai, H.; Jia, D.Z.; Su, Y.; Wang, T.; Zhou, W.Y. Three-dimensional Co_3O_4@$NiMoO_4$ core/shell nanowire arrays on Ni Foam for electrochemical energy storage. *Electrochim. Acta* **2015**, *180*, 998-1006.
4. Budhiraju, V.S.; Kumar, R.; Sharma, A.; Sivakumar, S. Structurally stable hollow mesoporous graphitized carbon nanofibers embedded with NiMoO4 nanoparticles for high performance asymmetric supercapacitors. *Electrochim. Acta* **2017**, *238*, 337-348.
5. Zhang, C.; Sui, Q.; Lu, L.; Zou, Y.; Xu, F.; Sun, L.; Cai, D.; Xiang, C. Hollow core-shell $CuCo_2O_4$@MoNi-layered double hydroxides as an electrode material for supercapacitors. *J. Energy Storage* **2023**, *61*, 106691.
6. Liu, S.; Hui, K.S.; Hui, K.N.; Yun, J.M.; Kim, K.H. Vertically stacked bilayer $CuCo_2O_4$/$MnCo_2O_4$ heterostructures on functionalized graphite paper for high-performance electrochemical capacitors. *J. Mater. Chem. A* **2016**, *4*, 8061-8071.
7. Hao, C.; Guo, Y.N.; Xian, S.L.; Zheng, W.H.; Gao, H.W.; Wang, X.H. Fabrication of flower-shaped $CuCo_2O_4$@$MgMoO_4$ nanocomposite for high-performance supercapacitors. *J. Energy Storage* **2021**, *41*, 102972.
8. Wan, L.; Liu, J.; Li, X.; Zhang, Y.; Chen, J.; Du, C.; Xie, M. Fabrication of core-shell $NiMoO_4$@MoS_2 nanorods for high-performance asymmetric hybrid supercapacitors. *Int. J. Hydrogen Energy.* **2020**, *45*, 4521-4533.
